# Convergent pathways in Parkinson’s disease

**DOI:** 10.1007/s00441-017-2700-2

**Published:** 2017-10-23

**Authors:** Marta Cherubini, Richard Wade-Martins

**Affiliations:** Oxford Parkinson’s Disease Centre, Department of Physiology, Anatomy and Genetics, Le Gros Clark Building, University of Oxford, South Parks Road, Oxford, OX1 3QX UK

**Keywords:** Parkinson’s disease, Dopamine neurons, Autophagy, Calcium, Intracellular trafficking

## Abstract

Preferential degeneration of dopamine neurons (DAn) in the midbrain represents the principal hallmark of Parkinson’s disease (PD). It has been hypothesized that major contributors to DAn vulnerability lie in their unique cellular physiology and architecture, which make them particularly susceptible to stress factors. Here, we report a concise overview of some of the cell mechanisms that may exacerbate DAn sensitivity and loss in PD. In particular, we highlight how defective protein sorting and clearance, endoplasmic reticulum stress, calcium dyshomeostasis and intracellular trafficking converge to contribute synergistically to neuronal dysfunction in PD pathogenesis.

## Introduction

**Fig. 1:**
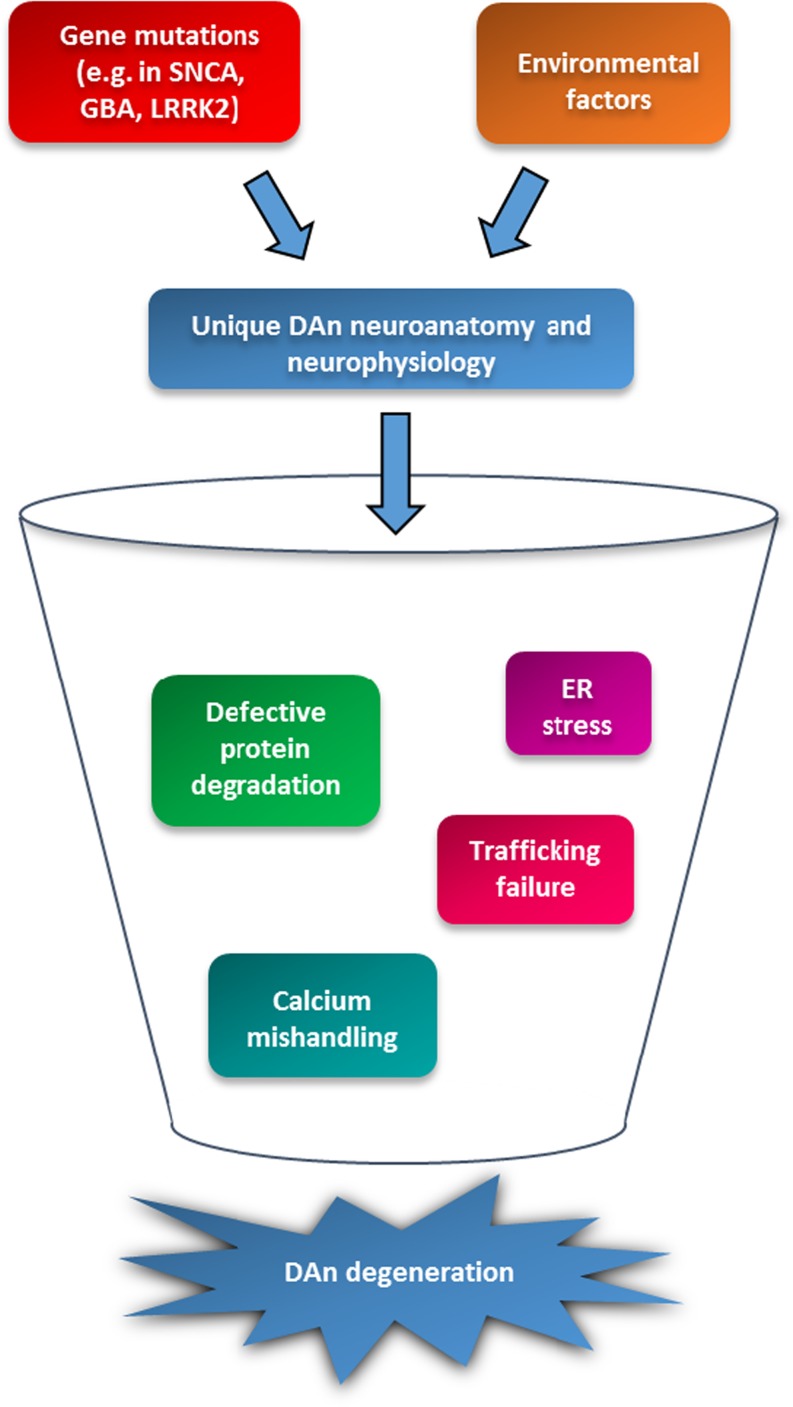
Convergent pathways towards dopaminergic neurodegeneration in PD. Combination of genetics and environmental factors exacerbate selective vulnerability of DAn by dysregulation of related cellular pathways including proteostatic dysfunction, ER stress, rupture of intracellular trafficking and alteration of calcium homeostasis

Parkinson’s disease (PD) is one of the most common age-related neurodegenerative disorders, characterized clinically by a progressive appearance of motor deficits that include resting tremor, muscular rigidity, bradykinesia, postural abnormalities and instability (Jankovic 2008). The underlying cause of PD is often attributed to an interplay between environmental and genetic factors (Horowitz and Greenamyre 2010); however, the majority of the cases are idiopathic and the underlying etiology remains largely elusive. Significant advances in understanding the mechanisms of disease pathogenesis have been made in the past two decades with the identification of pathogenic mutations associated with parkinsonism, although these genetic anomalies only account for 5–10% of PD patients (Corti et al. 2011). Whether it is the sporadic or hereditary form, the common feature is the preferential loss of dopamine neurons (DAn) of the substantia nigra (SN) projecting to the striatum (Damier et al. 1999). These neurons show unique cellular features that make them more susceptible than other neuronal populations in the brain. Indeed, their extensive and unmyelinated axonal innervation conveys an exceptionally high energy cost that makes them more vulnerable to any perturbation of energy supply (Bolam and Pissadaki 2012). Moreover, alteration of different cellular mechanisms such as protein degradation pathways, endoplasmic reticulum (ER) function, calcium signaling and intracellular trafficking enhance the vulnerability of DAn converging towards their progressive degeneration (Fig.1).

In this review, we discuss recent insights into the study of these aberrant mechanisms and we highlight how some of them share common elements finally contributing to the pathogenesis of PD.

## Dysfunctional autophagy/lysosome pathways in PD

Autophagy is a dynamic cellular homeostatic process essential for bulk degradation of cytoplasmic contents and constitutes the main proteolytic system in neurons (Nikoletopoulou et al. 2015). According to the pathway by which the autophagic cargo is delivered within the lysosomes, three types of autophagy can be distinguished: microautophagy, macroautophagy and chaperone-mediated autophagy (CMA) (Cuervo 2010). Microautophagy takes place when cytosolic components are directly internalized through invaginations at the lysosome membrane and, once in the lysosomal lumen, the whole internalized vesicle is degraded (Kunz et al. 2004). In contrast, during macroautophagy, the sequestration of cytosolic cargo is triggered by a double-membrane phagophore that expands into a vesicle called autophagosome. Subsequently, the autophagosome fuses with lysosomes to allow the cargo to be degraded and recycled (Klionsky 2005). CMA differs from the other two autophagic pathways since it does not involve vesicle formation and the degradation is based on the recognition of a specific amino acid sequence (KFERQ). In this case, the cargo, which is recognized by the cytosolic heat shock cognate protein 70 (Hsc70), directly translocates into the lysosome across the lysosomal membrane with the help of a set of lysosomal proteins such as the lysosome-associated membrane protein type 2A (LAMP2A) (Kon and Cuervo 2010).

**Fig. 2:**
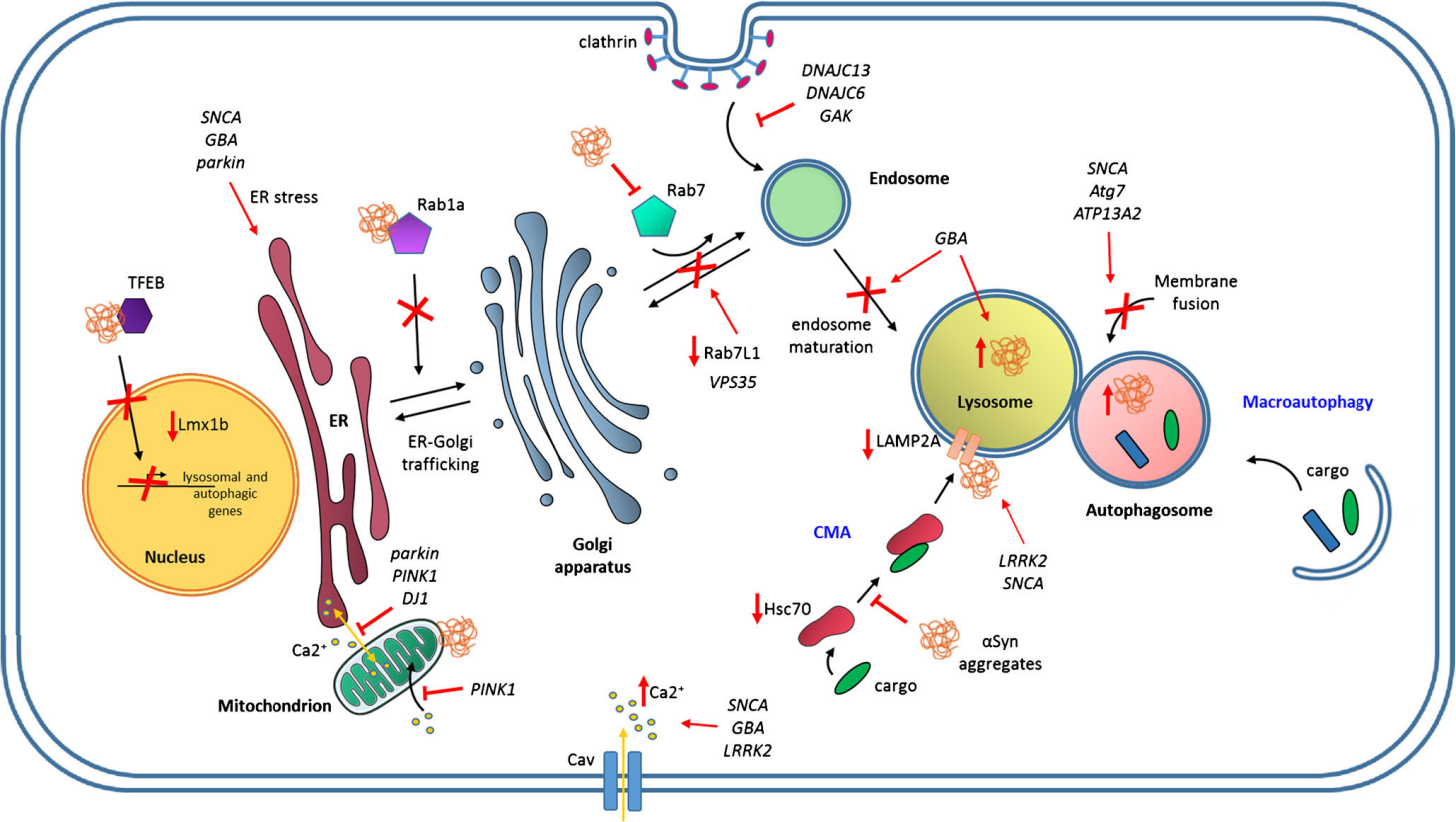
Dysfunctional cellular mechanisms in PD. Convergent molecular mechanisms in protein homeostasis, intracellular trafficking, the autophagic/lysosomal pathway and calcium signaling in Parkinson’s. Aggregation of misfolded αSyn is responsible for ER stress and failure of autophagic pathways and sequestration of Hsc70 into the lysosome. Impairment of macroautophagy machinery is attributable to mutations in *SNCA*, *Atg7*, *ATP13A2* and *LRRK2*. Association of αSyn transcription factor TFEB blocks TFEB translocation into the nucleus, hence impairing the expression of autophagy/lysosome-related genes. αSyn aggregates inhibit ER-Golgi vesicular transport by interaction with Rab1a and impair endosomal transport and fusion with lysosome by disrupting Rab7 function. PD-related mutations in *DNAJC13*, *DNAJC6* and *GAK* induce perturbation in endosomal trafficking and disrupt clathrin mediated endocytocis. Parkinson’s-associated mutations lead to Ca^2+^ mishandling and Ca^2+^ dyshomeostasis through alteration of ER-mitochondrial tethering, reducing Ca^2+^ transport between organelles and a failure of intracellular buffering mechanisms. Sustained Cav channel opening underlies low intrinsic buffering capacity dopaminergic neurons leading to elevated Ca^2+^ influx

Autophagy is generally considered to exert a neuroprotective role (Dohi et al. 2012; Rodriguez-Muela and Boya 2012; Jeong et al. 2013) and growing evidence indicates that its down-regulation leads to the accumulation of aberrant proteins as inclusion bodies contributing to the pathogenesis of neurodegenerative disorders (Hara et al. 2006; Komatsu et al. 2006). Indeed, as neuronal cells are more sensitive to accumulation of toxic components than dividing ones, high activity of the intracellular protein degradation systems appears to be crucial in order to maintain neuronal function and prevent cell death. The presence of intraneuronal protein inclusions within the brain stem (Spillantini et al. 1997) supports a role for autophagic failure in PD. These insoluble protein aggregates, known as Lewy bodies (LBs), are principally composed of α-synuclein (αSyn), a normally presynaptic protein with a physiological function related to neurotransmitter release at the nerve terminal (Bendor et al. 2013). In its native state, αSyn is typically unfolded (Weinreb et al. 1996); however, the protein is extremely sensitive to its environment and can change its conformation to monomeric and oligomeric states, leading to misfolding and aggregation during the process (Uversky 2007). CMA and macroautophagy are responsible for αSyn degradation and impairment of either of these two pathways has been related to its pathogenic accumulation in PD (Vogiatzi et al. 2008) (Fig. 2). CMA-related proteins, Hsc70 and LAMP2A, were found reduced in the SN and amygdala of post-mortem brain from PD patients (Alvarez-Erviti et al. 2010), providing additional evidence for altered CMA activity in PD. Moreover, LAMP2A reduction is correlated with increased αSyn and decreased levels of Hsc70 in the early stages of PD (Murphy et al. 2015), suggesting that CMA dysregulation occurs before substantial αSyn aggregation in PD. The involvement of dysfunctional macroautophagy in αSyn accumulation was demonstrated by the finding that specific deletion of the autophagy-related gene 7 (Atg7) results in accumulation of αSyn aggregates together with loss of dopaminergic neurons and reduced striatal dopamine content (Ahmed et al. 2012; Friedman et al. 2012). Accumulating evidence also suggests that αSyn and its pathogenic forms can exert inhibitory effects on the degradation pathways. For instance, αSyn overexpression can inhibit autophagosome biogenesis through interaction with the autophagy regulator Rab1a (Winslow et al. 2010). Association of αSyn with the transcription factor EB (TFEB), a critical regulator of lysosomal biogenesis, autophagy and lysosomal degradation, has been recently suggested as a mechanism underlying autophagy/lysosomal pathway dysfunction. Indeed, high cellular levels of αSyn in nigral DAn block the translocation of TFEB to the nucleus, leading to a depletion of markers of lysosome function (Decressac et al. 2013). In addition, a recent study reported that αSyn aggregates are able to resist macroautophagy clearance, leading to a failure of the pathway and accumulation of autophagosomes (Tanik et al. 2013). Furthermore, two αSyn familial mutations, *SNCA-A30P* and *SNCA-A53T*, have been involved in the impairment of the CMA pathway (Cuervo et al. 2004). Both mutant forms of the protein exhibited higher affinity to the LAMP2A receptor than the wild-type form but failed to be degraded, favoring toxic gains-of-functions by contributing to its aggregation or additional modifications, such as dopamine-adduct formation (Martinez-Vicente et al. 2008), which further underlie PD DAn vulnerability.

Besides αSyn, other PD-related genes have been shown to contribute to the autophagy failure. One of them is the leucine-rich repeat kinase 2 (LRRK2) in which autosomal-dominant mutations represent the major common monogenic forms of familial PD (Zimprich et al. 2004; Lubbe and Morris 2014). LRRK2 protein localizes in the cytosol as well as to specific membrane domains, including mitochondria and autophagosomes (Biskup et al. 2006; Alegre-Abarrategui et al. 2009). Given its widespread localization, LRRK2 has been associated with several cellular functions and signaling pathways, including mitochondrial function and vesicle trafficking together with endocytosis and autophagy (Alegre-Abarrategui and Wade-Martins 2009; Wallings et al. 2015). Recent findings have shown that two of the most frequent mutations of LRRK2, *G2019S* and *R1441C*, result in progressive degeneration of dopaminergic neurons due to the accumulation of autophagic vacuoles and increased mitochondrial autophagy (Ramonet et al. 2011). In addition, primary fibroblasts carrying these PD-related pathogenic mutations exhibit alterations in markers for autophagy/lysosomal function (Manzoni et al. 2013), highlighting a role for LRRK2 in the dysfunction of the autophagy/lysosomal pathway in PD. In accordance with these findings, it has recently been demonstrated that LRRK2 regulates lysosome size, number and function and that expression of PD-associated LRRK2 variants results in enlarged lysosomes and reduces the lysosomal capacity of the cell (Henry et al. 2015). Moreover, DAn differentiated from induced pluripotent stem cells (iPSCs) generated from PD patients carrying familial LRRK2 mutations showed accumulation of autophagic vacuoles that occurs after defective autophagosome clearance (Sanchez-Danes et al. 2012). Interestingly, this impairment in autophagy is associated with abnormal accumulation of αSyn, suggesting that the two proteins may act synergistically to induce neurodegeneration and that LRRK2 can accelerate mutant αSyn-induced neuropathology as previously reported in mice in a dose-dependent manner (Lin et al. 2009). Further evidence supporting this hypothesis comes from another study that reported that pathogenic LRRK2 may promote oligomerization of αSyn on the lysosomal surface, inhibiting its uptake into the lysosome and CMA-mediated degradation (Orenstein et al. 2013) (Fig. 2).

Autophagic perturbations with subsequent accumulation of αSyn have also been observed when glucocerebrosidase (GCase) activity is compromised. GCase is a lysosomal enzyme encoded by the GBA gene and mutations are recognized as an important genetic risk factor for the development of PD (Sidransky et al. 2009). In studies conducted in neurons derived from iPSC lines carrying either partial depletion of GCase or common mutated forms (*GBA-N370S* and *GBA-L444P*) demonstrate impairment of lysosomal protein degradation and a substantial increase of αSyn levels (Mazzulli et al. 2011; Schondorf et al. 2014; Fernandes et al. 2016). Importantly, experiments conducted in brain tissue from patients with sporadic PD confirm that GCase activity is also reduced without GBA mutations and is associated with lysosomal dysfunction and accumulation of αSyn (Murphy et al. 2014). Recently, it has been proposed that these detrimental events following GCase deficiency are due to the impairment of lysosomal recycling and endosome maturation processes (Magalhaes et al. 2016) (Fig. 2). Accordingly, mutations in other genes encoding proteins of endosomal/lysosomal processes such as the cation-transporting ATPase (ATP13A2) result in PD-like neuropathology through defective autophagy. The ATP13A2 gene (also known as PARK9) is mutated in autosomal recessive forms of early-onset Parkinsonism with pyramidal degeneration and dementia (Ramirez et al. 2006). Experiments conducted in ATP13A2 PD patient-derived fibroblasts showed that ATP13A2 loss of function leads to several lysosomal alterations including impaired lysosomal acidification, decreased proteolytic processing of lysosomal enzymes, reduced degradation of lysosomal substrates and diminished lysosomal-mediated clearance of autophagosomes (Dehay et al. 2012). Moreover, ATP13A2 deficiency drives neurotoxicity through the accumulation of αSyn (Usenovic et al. 2012). Importantly, recent findings highlight that ATP13A2 may be capable of regulating synaptotagmin 11 (SYT11), another protein emerging with a function in PD (Usenovic et al. 2012). Knockdown of ATP13A2 decreases SYT11 transcription, which in turn blocks autophagosome clearance and compromises lysosomal function (Bento et al. 2016). Thus, defects in the ATP13A2/SYT11 network are likely to contribute to lysosomal dysfunction, autophagy blockage and impairment of αSyn clearance associated with PD. Finally, Lmx1b has been recently identified as a crucial transcription factor involved in the maintenance of normal autophagic/lysosomal and intracellular transport functions and its dysfunction has been tightly linked to the onset of PD pathology (Laguna et al. 2015). Expression of the transcription factors, Lmx1a and Lmx1b, is known to be necessary for the development of mid-brain DA neurons (Yan et al. 2011). Laguna and collaborators found that their activity persists even after the neurons have matured. Moreover, they observed reduced levels of Lmx1b in the SN DA neurons of PD brain tissue (Fig. 2), together with an alteration in the autophagic/lysosomal pathway clearance systems followed by degenerative loss of SN DA neurons in Lmx1b-ablated animal models.

## Defective intracellular trafficking in PD

Intracellular protein trafficking has an important role in the biology of neuronal function and survival. Two major cellular pathways are responsible for shuttling vesicles transport proteins and lipids between the membrane-bounded compartments of the cell and its environment (Tokarev et al. 2009). First, in the exocytic pathway, proteins synthesized in the cytoplasm are translocated into the ER and, from here, membranous vesicles shuttle the cargo to the Golgi apparatus. In the last step, the Golgi apparatus sorts and ships the proteins to their final cellular destinations, such as the plasma membrane for constitutive and regulated secretion but also to endosomes and lysosomes, or back to the ER. Second, in the endocytic pathway, proteins and membrane are internalized at the plasma membrane by either clathrin-dependent or clathrin-independent endocytosis and delivered to the early endosome where sorting occurs. When the endosome has matured into a late endosome, it finally fuses with a lysosome that represents the degradative end-point at which the endosomal and autophagic pathways converge.

Recent findings point to faulty trafficking as contributing to the pathological accumulation and clearance of misfolded proteins, finally leading to dysfunction and degeneration of neurons and neural circuits (Wang et al. 2014). Importantly, advances in genetics and experimental discoveries have highlighted that defects in the intracellular trafficking machinery are involved in the development of PD (Hunn et al. 2015; Abeliovich and Gitler 2016), which may explain the association of the microtubule-associated membrane tau with PD. The high capacity of αSyn to bind to acidic phospholipid vesicles (Jo et al. 2000) suggests a role for αSyn in the regulation of vesicle trafficking and prompts the hypothesis that its pathogenic form may cause dysfunctional intracellular trafficking in PD. For instance, recent studies conducted in PD patient-derived iPSC neurons have shown that the accumulation of αSyn inhibits trafficking of important enzymes to the lysosome by altering the ER-Golgi localization of Rab1a, a key mediator of vesicular transport (Mazzulli et al. 2016) (Fig. 2). Interestingly, upregulation of Rab1 is sufficient to protect against αSyn-induced neuron loss, suggesting that enhancing protein trafficking can reverse the pathogenic link between αSyn and lysosomal dysfunction (Cooper et al. 2006; Mazzulli et al. 2016). Other evidence comes from studies conducted in primary neurons that demonstrated that αSyn aggregates impair Rab7-positive endosomal transport and fusion with lysosomes (Volpicelli-Daley et al. 2014). The familial PD-associated *SNCA-A53T* mutation in the gene encoding αSyn has also been associated with defects in the ER–Golgi transport, through the inhibition of the fusion of ER vesicles to the Golgi membrane (Gitler et al. 2008; Thayanidhi et al. 2010). The proposed mechanism reveals that *A53T* αSyn directly binds to ER–Golgi SNAREs, a class of proteins essential for the fusion of vesicles with membranes. This interaction is sufficient to inhibit SNARE complex assembly, reducing the events that lead to membrane fusion and suggesting a potential αSyn-dependent toxic effect on synaptic vesicle exocytic machinery. Importantly, a study conducted in A53T-αSyn transgenic mice showed that *A53T* αSyn-induced ER–Golgi trafficking defects can be exacerbated by the overexpression of LRRK2, suggesting a synergistic cytotoxic effect that finally leads to the fragmentation of the Golgi apparatus and increased αSyn accumulation in the soma (Lin et al. 2009).

Co-localization of LRRK2 with the late endosomal marker Rab7 in αSyn-positive brainstem LBs implicates LRRK2 in the function of the endo-lysosomal pathway (Higashi et al. 2009). Notably, the expression of mutant LRRK2 in both cell and animal models leads to defective late endosome maturation and fusion with lysosomes by impairing the interaction with Rab7 and its function (Dodson et al. 2012; Gomez-Suaga et al. 2014). Recent phosphoproteomic screens have revealed that one of the key functions of LRRK2 kinase activity is to regulate the activity of proteins from the Rab family and, consequently, vesicular trafficking events (Steger et al. 2016). Indeed, apart from Rab7, LRRK2 has been shown to interact with other Rab proteins including Rab29/Rab7L1, a Golgi-resident Rab encoded by the PARK16 non-familial PD risk-associated locus (MacLeod et al. 2013). In this study, it was reported that knockdown of Rab7L1 recapitulated degeneration observed with the expression of a familial PD mutant form of LRRK2 in rodent or *Drosophila* dopamine neurons, whereas Rab7L1 overexpression rescued the LRRK2 mutant phenotypes. This neuronal loss is attributable to defective endo-lysosomal and Golgi apparatus sorting defects (Fig. 2). Interestingly, these defects can be rescued by the expression of wild-type VPS35, a component of the retromer complex, which mediates endosome–Golgi retrieval of membrane proteins (Bonifacino and Hurley 2008). Recently, it has been shown that a PD-causing mutation of VPS35 protein induces marked degeneration of dopaminergic neurons (Tsika et al. 2014; Tang et al. 2015) and that defects in autophagy, as well as in the trafficking of lysosomal protein cathepsin D and the transmembrane autophagy protein ATG9A, have also been proposed as putative mechanisms (Follett et al. 2014; Zavodszky et al. 2014). Indeed, mutant VPS35 exhibits reduced association with the WASH complex (McGough et al. 2014), impairing its endosomal recruitment and thus perturbing endosomal/lysosomal trafficking.

Recent investigations on further genes linked to PD have underscored the importance of the endocytic pathway in disease pathogenesis. Among them, mutations in *DNAJC13*, *DNAJC6* and *GAK*, which encode for functionally related proteins that control clathrin-dependent endocytosis, have been associated with familial and sporadic PD (Fig. 2). In particular, the receptor-mediated endocytosis 8/RME-8 (*DNAJC13*) regulates the dynamics of clathrin coating on early endosomes and recent studies have reported that the p.Asn855Ser mutation confers a toxic gain-of-function and impairs endosomal transport (Vilarino-Guell et al. 2014). Similarly, mutation in auxilin 1 encoded by *DNAJC6* leads to impaired synaptic vesicle recycling and perturbed clathrin-mediated endocytosis by increased retention of assembled clathrin on vesicles and in empty cages (Edvardson et al. 2012). Cyclin G-associated kinase (GAK) plays key roles for clathrin exchange as well as for clathrin uncoating (Lee et al. 2006) and single nucleotide polymorphisms (SNPs) in the *GAK* locus have been identified as risk factors for sporadic PD by genome-wide association studies (Nalls et al. 2014). Especially, microarray analysis of post-mortem PD and control brains has demonstrated a significant correlation between one of the identified *GAK* SNPs and increased αSyn expression (Dumitriu et al. 2011). This event is recapitulated when GAK expression is downregulated in cell culture, causing a significant increase in toxicity. Intriguingly, GAK has also been proposed to bind and form a complex with LRRK2 with the function of promoting the clearance of Golgi-derived vesicles (Beilina et al. 2014). Altogether, these genetics findings suggest that variants in different loci converge towards defective trafficking and sorting, culminating in lysosomal dysfunction and aberrant protein degradation.

## ER stress in PD

It is well established that ER stress is also a potent trigger of autophagy and the occurrence of chronic ER stress has been extensively described in neurodegenerative conditions, including PD (Ogata et al. 2006; Matus et al. 2008). Importantly, ER stress and autophagy cooperate to protect cells by relieving stress and inducing cell death under extreme conditions, thus alteration of the function of one of these systems can influence the other (Rashid et al. 2015).

The ER is a sensitive sensor of cellular homeostasis and maintains proper protein folding and quality control (McCaffrey and Braakman 2016). Disruption of the folding process and accumulation of misfolded or unfolded proteins in the ER trigger the activation of the unfolded protein response (UPR) to counteract the situation (Hetz 2012). Three main stress sensors recognize unfolded proteins in the ER, including inositol-requiring protein-1 (IRE1), protein kinase RNA-like ER kinase (PERK) and the activating transcription factor-6 (ATF6). The activation mechanism of these proteins has not been completely defined but it is known that molecular chaperones of ER lumen, such as BiP (Grp78), are involved in the activation of these transmembrane transducers (Lee 2005). Many reports suggest the involvement of ER stress in the pathology of PD and accumulation of misfolded αSyn has been proposed as a major cause of this deleterious process. For instance, expression of A53T-αSyn in differentiated PC12 cells showed decreased proteasome activity and increased ER stress (Smith et al. 2005). In another recent study, αSyn overexpression and accumulation inhibited the neuroprotective activity of ATF6, which is normally processed via COPII-mediated ER–Golgi transit following its activation via ER stress (Credle et al. 2015). Lower levels of ATF6 and its reduced incorporation in COPII vesicles were also observed in presence of mutated A53T-αSyn. Impaired ATF6 signaling is accompanied by decreased ER-associated degradation (ERAD) function, which sensitizes cells to apoptosis, thereby disrupting UPR signaling. However, how αSyn could initiate ER stress is still a matter of debate. It has been suggested that αSyn may induce ER stress by disrupting ER–Golgi vesicular trafficking leading to ER overload (Cooper et al. 2006). Moreover, αSyn toxic oligomers may accumulate in the lumen of the ER early during the disease process, with subsequent upregulation of ER chaperones (Colla et al. 2012). Furthermore, activation of the PERK–eIF2α pathway of the UPR occurs concomitantly with αSyn cytoplasmic accumulations in nigral dopaminergic neurons of PD cases (Hoozemans et al. 2007). Increase of the UPR mediators and ER stress also occurs when misfolded GCase is trapped in the ER (Ron and Horowitz 2005). In line with this, iPSC-derived DA neurons carrying the PD-associated *GBA-N370S* mutation showed activation of UPR with significant upregulation of ER-resident chaperones, such as BiP, IRE1α and PDI (Fernandes et al. 2016). Furthermore, evidence has highlighted the pathogenic role of parkin in regulating the ERAD of misfolded ER proteins. Mutations of the parkin gene, which compromise the ubiquitin ligase function of the protein (Dawson and Dawson 2010), result in the accumulation of its substrates in the ER of SN dopaminergic neurons, leading to ER stress and cell death (Imai et al. 2001).

Apart from the ER stress response associated with aggregation of misfolded proteins, it has been recently shown that downregulation of parkin may increase the vulnerability of cells to ER stress-induced mitochondrial dysfunctions, suggesting an interconnection between mitochondrial and ER stress, with parkin playing a central role in this connection (Bouman et al. 2011) (Fig. 2). In line with this, mutations in parkin and PTEN-induced putative kinase 1 (PINK1) may enhance ER stress signaling through defective ER-mitochondria tethering (Celardo et al. 2016). This deleterious event is due to an increase in contacts between mitochondria and the ER, which is promoted by increased levels of the profusion factor mitofusin. Thus, activation of ER stress seems to be linked to the functional status of mitochondria at ER contacts and disruption of these connections may enhance the vulnerability of DAn in PD. Interestingly, point mutations in αSyn can reduce the number of appositions between ER and mitochondria, thereby altering their function (Guardia-Laguarta et al. 2014). Moreover, downregulation of DJ-1, a protein associated with rare forms of inherited early-onset PD, promotes morphological changes in mitochondria, leading to alteration of the contacts between the two organelles (Ottolini et al. 2013). Impairment of ER–mitochondria tethering finally reduces calcium transfer between the two compartments, indicating the importance of these interactions in order to preserve normal physiology and prevent neurodegeneration.

## Calcium signaling in PD

Calcium (Ca^2+^) serves multiple complex and integrated functions in neurons, including the control of dendritic responses to neurotransmitters, signaling to the nucleus to regulate gene expression and initiation of neurotransmitter release from presynaptic axon terminals (Brini et al. 2014). Ca^2+^ is a messenger that transfers signals within the cell in response to membrane depolarization, thereby relaying information on neuronal activity status within the neuron. Ca^2+^ signals are decoded based on the characteristics of the intracellular changes in Ca^2+^ concentration and generate outputs as different as proliferation or death. Owing to its vital importance, a coordinated system to control Ca^2+^ concentration is required to guarantee proper neuronal function. This system includes Ca^2+^-buffering proteins, exchangers, pumps and voltage- and ligand-gated channels in the plasma membrane that regulate active extrusion of the ion to the extracellular space or sequestration into intracellular organelle stores. Neurons express various types of voltage-gated Ca^2+^ (Cav) channels including the Cav1 or L-type, Cav2 and Cav3 isoforms (Hurley and Dexter 2012). The Cav1 and Cav2 channels activate at high depolarization voltage and produce sustained large Ca^2+^ currents, whereas the Cav3 channels activate at low voltage, generating transient Ca^2+^ currents. Importantly, SN dopaminergic neurons differ from many other neuronal populations by having autonomous activity and generating continuous low frequency (2–10 Hz) activity in the absence of conventional synaptic input (Grace and Bunney 1983). This phenomenon is dependent on Cav1-type channels, especially on the pore-forming Cav1.3 subunit, which opens at more hyperpolarized potentials than Cav1.2 channels, thus exposing SN dopaminergic neurons to elevated Ca^2+^ influx (Xu and Lipscombe 2001; Chan et al. 2007) (Fig. 2). Although this autonomous pacemaking activity serves to maintain a constant dopamine supply in the striatum (Surmeier 2007), it may underlie the preferential vulnerability of SN DA neurons that exhibit low intrinsic Ca^2+^-buffering capacity (Foehring et al. 2009), in part due to low levels of the protein calbindin. Indeed, recent work has shown that continuous rises in cytosolic Ca^2+^ occurring in SN DA neurons are able to induce mitochondrial oxidative stress (Guzman et al. 2010). Oxidant stress generates mild mitochondrial depolarization or uncoupling, which leads to bioenergetic deficiency, exacerbating the vulnerability of DAn towards a condition of increased metabolic demand. Increased vulnerability to stress responses involving elevation of cytosolic Ca^2+^ overload was also observed in iPSC-derived neurons harboring GBA mutations (Schondorf et al. 2014). Dysregulation of calcium homeostasis in GBA mutant neurons was accompanied by an increased expression of the neuronal calcium-binding protein NECAB2, suggesting a compensatory mechanism in such vulnerable neurons. Defects in Ca^2+^-buffering capacity are also evident in neurons expressing mutant LRRK2. Several studies support a role for LRRK2 in Ca^2+^ signaling and homeostasis, reporting that mutated forms of the kinase protein generate altered calcium levels along with aberrations in mitochondrial degradation, dendrite shortening and neurite aggregation (Cherra et al. 2013; Schwab and Ebert 2015). In line with this, it has been recently demonstrated that LRRK2 can impact Cav channel function, especially Cav of type 2 (Bedford et al. 2016). Interestingly, the authors found that CaV2.1 activation is dependent on the kinase activity of LRRK2 causing a large increase in Ca^2+^ current. Moreover, the PD-related G2019S LRRK2 mutation stimulated CaV2.1 channels to a greater degree than the wild-type, supporting the hypothesis that LRRK2 mutations disrupt normal Ca^2+^ signaling in PD. In addition to LRRK2, other studies have pointed out that αSyn can alter calcium homeostasis, enhancing the voltage-operated Ca^2+^ channel activity. Indeed, the incubation of differentiated neuroblastoma cell lines and primary rat cortical neurons with a medium containing secreted αSyn induces an increase in capacitive Ca^2+^ entry and calpain-mediated toxicity (Melachroinou et al. 2013). Accordingly, dysregulation of spontaneous and stimulus-evoked neuronal calcium activity was observed in transgenic mice overexpressing human αSyn (Reznichenko et al. 2012). The augmented Ca^2+^ transients and defective decay of the Ca^2+^ peak without any change in the neuronal spiking response suggest that αSyn promoted alterations in Ca^2+^ dynamics via interference with intracellular buffering mechanisms.

Due to their capacity to buffer high cytosolic calcium levels, functional mitochondria are crucial in order to prevent Ca^2+^ dyshomeostasis. Evidence for a possible role of mitochondrial Ca^2+^ mishandling in the pathogenesis of PD comes from studies on the mitochondrial kinase, PINK1. The first suggestion arose from the finding that the expression of mutant PINK1 exacerbates mitochondrial defects in a cellular model of PD. These defects, such as loss of mitochondrial membrane potential, increased mitochondrial size with loss of cristae and reduced ATP levels, are fully rescued by the inhibition of the mitochondria calcium uniporter, suggesting that mitochondrial Ca^2+^ uptake is involved (Marongiu et al. 2009). Other studies have proposed that the absence of PINK1 induces mitochondrial Ca^2+^ accumulation, possibly as a consequence of the impairment of mitochondrial Ca^2+^ efflux through the mitochondrial Na^+^/Ca^2+^ exchanger (Gandhi et al. 2009). Depletion of PINK1 could also impair mitochondrial Ca^2+^ uptake and, consequently, ATP production (Heeman et al. 2011) (Fig. 2). Moreover, increased sensitivity of mitochondria to Ca^2+^-induced permeability has been shown to precede dopaminergic defects in PINK1-deficient mice, suggesting that mitochondrial Ca^2+^ alteration could be an early event in the pathogenesis of PD (Akundi et al. 2011). Growing evidence also supports a role for aSyn in modulating mitochondrial Ca^2+^ fluxes. Experiments in neuroblastoma cells overexpressing A53T mutant aSyn demonstrated that aSyn localizes at the mitochondrial membrane and that its accumulation was directly related to an increase of intramitochondrial Ca^2+^ (Parihar et al. 2008). In line with these findings, another study reported that oligomeric αSyn disrupts mitochondrial function leading to complex I dysfunction, altered membrane potential, disrupted Ca^2+^ homeostasis and enhanced cytochrome c release (Luth et al. 2014). A possible mechanism recently described supports a role for αSyn in the perturbation of ER-mitochondria associations (Paillusson et al. 2017) (Fig. 2). Indeed, αSyn in PD iPSC-derived neurons and cellular models alters ER–mitochondrial Ca^2+^ exchange and reduces mitochondrial ATP production by disruption of the VAPB-PTPIP51 tethering proteins. Therefore, the physical link between ER and mitochondria appears to be crucial to regulate Ca^2+^ accumulation by mitochondria in order to maintain the energetic supply in these neurons and ultimately preserve their survival.

## Conclusions and future perspectives

Here, we reviewed the growing evidence suggesting that the preferential demise of SN dopaminergic neurons that characterizes PD results from a combination of cellular insults and the intrinsic susceptibility of DAn arising from their physiology and anatomy. Due to the multifactorial complexity of PD pathogenesis, it is increasingly important to understand and elucidate the cellular mechanisms involved and how they converge, leading to the decline in neuronal function.

Considerable progress in our understanding of cellular pathways in PD pathogenesis came first from the genetic studies that identified genes linked to inherited forms of the disease and risk loci observed associated with the sporadic forms (Labbe and Ross 2014; Nalls et al. 2014). An important goal for those working on the molecular mechanisms of Parkinson’s is to link these genetic findings with cell biology to identify common pathways and themes and, finally, determine shared cellular targets for therapeutic intervention. In our review, we brought together the four themes that we see as central to Parkinson’s: the autophagic/lysosomal pathway, ER stress, intracellular trafficking and calcium signaling. Central to these pathways and at the point of convergence, is lysosomal dysfunction, which we consider a leading current target for therapeutic intervention across Parkinson’s.

The rapid accumulation of new systems-level data requires the development of new approaches to organize and analyze the extensive information on this field. The creation of molecular interaction maps has started to facilitate the detection of integrative pathways and the prioritization of specific targets for further investigation (Kanehisa et al. 2010; Fujita et al. 2014). The emergence of several -omics techniques, including transcriptomics, proteomics and metabolomics, has not only confirmed previously identified pathways associated with DAn degeneration but has also enhanced the ability to identify novel pathways and biomarkers related to PD pathogenesis (Caudle et al. 2010; Ren et al. 2015). Finally, the combination of gene expression data from transcriptomic analysis of patient cells with databases for the Connectivity Map, a technique that analyzes the transcription patterns produced by different chemicals, provides a new opportunity for the identification of potential novel therapeutic compounds for this disorder (Sandor et al. 2017).
